# Renal protection: a leading mechanism for cardiovascular benefit in patients treated with SGLT2 inhibitors

**DOI:** 10.1007/s10741-020-10024-2

**Published:** 2020-09-08

**Authors:** Davide Margonato, Giuseppe Galati, Simone Mazzetti, Rosa Cannistraci, Gianluca Perseghin, Alberto Margonato, Andrea Mortara

**Affiliations:** 1 Heart Failure Unit and Department of Cardiology, Policlinico di Monza, Via Amati 111, 20900 Monza, Italy; 2 Department of Cardiology, Fondazione IRCCS Policlinico San Matteo, Pavia, Italy; 3 Heart Failure Unit and Department of Cardiology, San Raffaele Hospital and Scientific Institute (IRCCS), Milan, Italy; 4 Department of Medicine and Surgery, Università Degli Studi di Milano Bicocca, & Policlinico di Monza, Monza, Italy

**Keywords:** SGLT2 inhibitors, Chronic kidney disease, Type 2 diabetes mellitus, Renal protection, Cardiovascular outcomes, Heart failure

## Abstract

Initially developed as glucose-lowering drugs, sodium-glucose co-transporter type 2 inhibitors (SGLT2i) have demonstrated to be effective agents for the risk reduction of cardiovascular (CV) events in patients with type 2 diabetes mellitus (T2DM). Subsequently, data has emerged showing a significant CV benefit in patients treated with SGLT2i regardless of diabetes status. Renal protection has been initially evaluated in CV randomized trials only as secondary endpoints; nonetheless, the positive results gained have rapidly led to the evaluation of nephroprotection as primary outcome in the CREDENCE trial. Different renal and vascular mechanisms can account for the CV and renal benefits enlightened in recent literature. As clinical guidelines rapidly evolve and the role of SGLT2i appears to become pivotal for CV, T2DM, and kidney disease management, in this review, we analyze the renal effects of SGLT2, the benefits derived from its inhibition, and how this may result in the multiple CV and renal benefits evidenced in recent clinical trials.

## Introduction

Sodium-glucose co-transporter type 2 inhibitors (SGLT2i) were originally developed to reduce hyperglycemia in diabetic patients via an insulin-independent mechanism, through their glycosuric effect. However, over the past 5 years, evidence from randomized clinical trials (RCTs) showed an unexpected benefit and safety in most cardiovascular (CV) and renal outcomes, irrespective on their impact on glycemic control.

Chronic kidney disease (CKD) occurs in approximately 40% type 2 diabetes mellitus (T2DM) patients [1] and is associated with a very high risk of CV diseases [2]. Moreover, heart failure (HF) and CKD frequently coexist, sharing diabetes as one of the main risk factors, thus interacting in a vicious circle which contributes to a poor prognosis [3]. The growing evidence from many RCTs on SGLT2i as a CV protective class of drugs led to a class 1 level A recommendation for their use in patients with T2DM and established atherosclerotic CV disease (ACVD), or in patients with T2DM and multiple risk factors but without an ACVD [4]. More recently, SGLT2i showed to significantly reduce morbidity and mortality even in HF irrespective of diabetes status [4, 5]^.^

Specific nephroprotection as a primary adjusted outcome has been evaluated only recently in the CREDENCE trial [6]; however, the mechanisms leading to renal benefit in patients treated with SGLT2i are still under debate.

This review focuses on the role of SGLT2i on kidney function, in order to clarify their potential direct and indirect benefits in preventing or delaying kidney damage, and on how this could translate into a reduction of CV and renal events as evidenced in recent trials.

## The role of SGLTs in the kidney

**Fig. 1:**
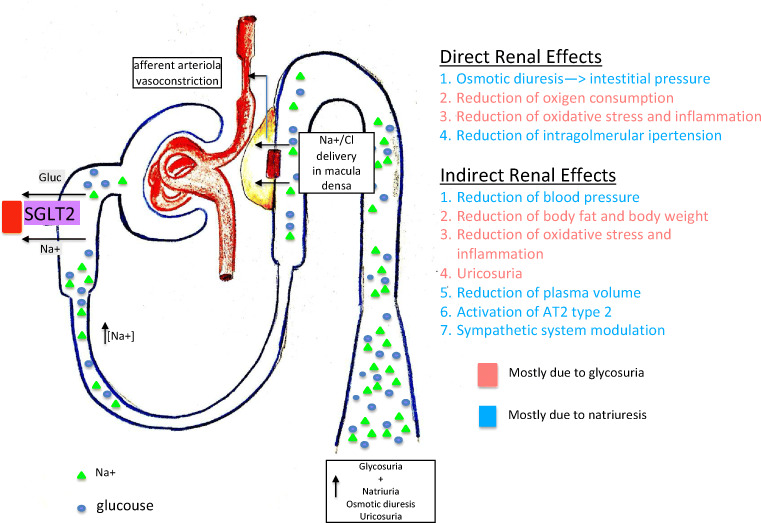
Effects of SGLT2 inhibition on the kidney and direct and indirect renal benefits obtained through SGLT2 inhibition (see in the text from reference 7 to reference 8)

Sodium-glucose transporters (SGLTs) are cell-membrane symporters that transfer sodium, together with glucose, into the cell down and against the concentration gradient, respectively [7] (see Fig. 1). SGLT2 is a high-capacity low-affinity transporter located in the first segment of the proximal tubule and is responsible for the reabsorption of about 90% of filtered glucose. Residual glucose is reabsorbed by the high-affinity low-capacity transporter SGLT1 in the distal segment of the proximal tubule [9]. In an individual with preserved glomerular filtration rate (GFR) and normoglycemia, all the glucose filtered in the proximal tubules is reabsorbed; conversely, an increase in the concentration of plasma glucose leads to a constant increase in filtered glucose, until the threshold of reabsorption is reached (normally for values of glycemia around 180–215 mg/dl) and glycosuria begins [10]*.*

Diabetes mellitus is associated with an improved capacity of renal glucose reabsorption probably through an increased activity of SGLT2 in the proximal tubules, sustaining hyperglycemia in a sort of vicious cycle [11]. Whether this increased expression of SGLT2 is a result of persistent exposure to hyperglycemia is still unclear [10, 12].

It is important to underline that, although SGLT2 has the main quantitative role in tubular glucose reabsorption, the benefit of SGLT2i may be slightly blurred by the concomitant activity of SGLT1. However, a double block on SGLTs could lead to both an increase in glycosuria and an enhanced risk of hypoglycemic events.

## Direct renal benefits

There are different direct effects of SGLT2i on kidney homeostasis that can explain the favorable renal outcomes reported in the literature. Firstly, SGLT2i, by reducing sodium reabsorption at the proximal tubules, causes an increase of sodium concentration at the macula densa, which in turn enhances sodium entrance in the cell and therefore its osmolarity [13]. The net effect is an increase in ATP’s conversion to adenosine leading to vasoconstriction of the afferent arterioles—via the tubuloglomerular feedback—and to a reduction of GFR. This process is of paramount importance since it reduces glomerular hyperfiltration, intraglomerular pressure, and consequently barotrauma and proteinuria, which are typical events of the early stage of diabetic nephropathy and HF, thus slowing the progression of nephropathy [14, 15].

Osmotic diuresis induced by SGLT2i is particularly relevant in the situation of interstitial volume overload working in synergy with the other diuretics, in particular loop diuretics [16]. Indeed, it has been demonstrated that SGLT2i primarily reduces interstitial volume, with a minor effect on intravascular volume [16, 17], while loop diuretics mostly reduce intravascular volume. This synergistic effect of SGLT2i and loop diuretics on both intravascular and interstitial volumes is very useful in states of volume overload by also limiting adverse effects of other diuretics such as inappropriate reflex neurohormonal stimulation, a response to intravascular volume depletion, and uric acid levels increase [18, 19]. SGLT2i give a significant advantage particularly in HF because they reduce the need for the first introduction of a loop diuretic—as shown in EMPA-REG-Outcome—limiting the renin-angiotensin-aldosterone system (RAAS) activation [20, 21]^.^

SGLT2i also mitigates the direct kidney damage with a suppression of numerous pathways linked to tubular hypoxia and fibrosis, such as oxidative stress, and inflammasome activity [22–24] regardless of diabetes [25] together with a proven significant reduction of urinary excretion of inflammatory markers [26].

## Indirect renal benefits

### Sympathetic nervous system

Several systemic mechanisms can in part account for the nephroprotective properties of SGLT2i. Elevated sympathetic activity is widely known as a major risk factor for renal disease [27, 28] and a known feature of diabetic patients [29]. The cross-talk between the sympathetic nervous system and SGLT2 has been proved with compelling evidence in animal models: in fact, it has been demonstrated that chemical sympathetic denervation in neurogenic hypertensive mice resulted in reduced renal SGLT2 expression, and that dapagliflozin-treated mice showed a significant decrease in the expression of markers of sympathetic activity and a reduction in blood pressure [30, 31]. The modulation of RAAS by SGLT2i has not been fully clarified. It has been suggested that this class of drugs activates the non-classic pathway of RAAS by stimulating the type 2 angiotensin II receptor with vasodilatation and anti-inflammatory properties, instead of the classic pro-pathogenetic pathway via the type 1 angiotensin II receptors [32]*.*

### Vascular protection

The micro- and macrovascular protection derived from sustained control of glycemia, blood pressure, and lipid profile has been widely recognized also for SGLT2i [33]. Hypertension is independently associated with the risk of developing future complications in T2DM, and the positive impact of lowering blood pressure in T2DM seems even more important than treating hyperglycemia [34, 35]*.* A reduction of systolic and diastolic blood pressure of 3–5 mmHg and 2–3 mmHg, respectively, has been reported with SGLT2i, both in monotherapy and in combination with other antidiabetic agents [36–38]*.* Natriuresis, a decrease in stress on vascular wall, and a reduction in body weight are the most important anti-hypertensive–related mechanisms [39–41]. Nonetheless, small reduction of blood pressure seems to be associated with minor renal benefit in the literature: in particular, as a reduction in albuminuria has been showed, favorable data on hard renal outcomes are controversial [42] but more recently emerging in the literature [43, 44]*.*

### Weight loss

A high body mass index (BMI) is one of the strongest risk factors for new-onset CKD [45], and the links between obesity and CKD are numerous and bidirectional [8]. Treatment with SGLT2i is accompanied by consistent and sustained weight loss, mainly caused by a reduction in visceral adipose tissue [46–48]*.* Decreased insulin secretion, increased urinary glucose excretion, increased lipolysis, and fat oxidation are metabolic effects induced by SGLT-2 inhibition that seem to significantly contribute to weight loss [49].

### Cardiac benefit

It is important to underline that the direct benefit of SGLT2i on the myocardium, especially in patients with HF or with an established cardiac disease, has beneficial effects also on kidney function and on renal outcomes. Direct effects on the myocardium are summarized in four main areas: reduction of left ventricular (LV) hypertrophy and mass and cardiomyocyte apoptosis; improvement of myocardial energetics and metabolomics; improvement of myocardial and ECM remodeling; reduction of myocardial inflammation and cytokines levels [21].

Finally, it is worth underlining that as the inhibition of SGLT2 is not insulin-dependent, the derived renal benefits abovementioned can be obtained during any phase of the natural history of diabetes, and the only limiting factor could be a severely reduced glomerular filtration rate, as in the end-stage renal disease (ESRD).

## Renal outcomes in RCTs on SGLT2 inhibitors: state of the art

In 2008, the Food and Drug Administration requested, as mandatory, the assessment of CV safety outcomes in RCTs evaluating new glucose-lowering therapies. Since then, a large amount of evidence has emerged pointing toward a CV safety and efficacy of novel antidiabetic drugs. In particular, beneficial effects of SGLT2i on CV outcomes have been recently demonstrated by empagliflozin [50], canagliflozin [51], and dapagliflozin [52] in patients with established ACVD and in those with multiple risk factors but without an established ACVD.

Renal protection as a primary endpoint by SGLT2i treatment was evaluated only in the most recent CREDENCE trial [6]: nonetheless, data on the effects of SGLT2 on kidney function has been reported from secondary or exploratory endpoints (EPs) in CV trials (see Table 1).

**Table 1:** Summary of renal data from renal and CV trials with SGLT2 inhibitors (specific references from the text are mentioned)

	Patients (n.)	Drug	Mean FU (years)	Mean eGFR	Renal endpoint	HR/*p* value
EMPA-REG OUTCOME [50]	7020	Empagliflozin	3,1	74.2 ml/min/1.73 m^2^	Rate of renal replacement therapy	*p* = 0.004
CANVAS Trial [51]	10,142	Canaglflozin	2,4	76.7 ml/min/1.73 m^2^	40% reduction in eGFR, death from renal cause or the need for renal replacement therapy	HR 0.6 95% IC 0.47–0.77)
DECLARE-TIMI 58 [52]	17,160	Dapagliflozin	4.2	85.4 ml/min/1.73 m^2^	Decrease in eGFR by at least 40%, CV or renal death and ESRD	HR 0.76 *p* < 0.001
DAPA-HF [5]	4744	Dapagliflozin	1,6	66.0 ml/min/1.73 m^2^	Sustained ≥ 50% reduction in eGFR, ESRD, or death from renal causes	HR 0.71 CI 0.44–1.16 *p* = 0.17
CREDENCE trial [6]	4401	Canagliflozin	2,6	56.3 ml/min/1.73 m2	Composite of ESRD (dialysis, transplantation, or a sustained eGFR less than 15 ml/min/1.73 m^2^), a doubling of the serum creatinine level, or death from renal or cardiovascular cause	HR 0.70; 95% CI 0.59–0.82, *p* = 0.00001
Zelniker et al. meta-analysis [53]	34,322				Reduced the risk of renal disease progression	HR 0.55 IC 0.48–0.64

In the 7020 patients with T2DM and established ACVD of the EMPA-REG OUTCOME [50], empagliflozin showed a significant 14% relative risk reduction (RRR) of the primary composite outcome (CV death, non-fatal myocardial infarction, or non-fatal stroke) driven by a 38% RR reduction of CV mortality (3.7% vs. 5.9%; hazard ratio [HR] 0.62; 95% confidence interval [CI] 0.49–0.77, *p* < 0.001) and a 35% RR reduction of hospitalizations for HF (2.7% vs. 4.1%, HR 0.65; 95% CI 0.5–0.85, *p* = 0.002) which was consistent across predefined subgroups, including patients with and without a previous history of HF. The placebo and the empagliflozin pooled group had similar baseline eGFR (73.8 ± 21.1 ml/min/1.73 m^2^ vs 74.2 ± 21.6 m/min/m^2^), but secondary renal EPs were significantly improved by empagliflozin. There was a 39% reduction of the established renal outcome such as progression of albuminuria, doubling of serum creatinine or ESRD, or renal death [54]. The rate of renal replacement therapy (RRT) was also lower in patients treated with empagliflozin compared with placebo (0.3% vs 0.6%, HR 0.45, 95% CI 0.21–0.97, *p* = 0.04). In summary, in this trial, treated patients presented a significantly lower risk of CV events, but also of acute kidney injury compared with the placebo group [55]. Subsequently, it was shown that a major effect of empagliflozin consisted in an acute reduction of eGFR and albuminuria, followed by long-term stability and preservation of eGFR, compared with the doubling of eGFR deterioration in the placebo group over the same period of time (3.1 years of median follow-up) [56]. Renal benefits provided by empagliflozin was observed even in patients with compromised renal function, independently from baseline HbA1c and achieved regardless of concomitant medications interacting with renal function [57, 58].

The CANVAS trial [51] enrolled 10,142 T2DM patients, 66% with an established ACVD and 34% with multiple risk factors without an established ACVD. Canagliflozin showed a 14% RR reduction of the primary composite outcome (the same of the EMPA-REG) versus placebo, but with a significant effect only on HF hospitalizations (33% RRR; 5.5% vs. 8.7%; HR 0.67; 95% CI 0.52–0.87), independently of the previous history of HF [51].

Even in the CANVAS trial, mean eGFR was similar in the placebo and canagliflozin groups (76.2 ± 20 ml/min/1.73 m^2^ vs 76.7 ± 20.3 ml/min/1.73 m^2^). The secondary endpoint of a sustained 40% reduction in eGFR, death from renal cause, or the need for renal replacement therapy (RRT) was significantly reduced by canagliflozin (HR 0.60; 95% CI 0.47–0.77) [59]. Interestingly, the effect on CV and renal outcomes did not differ according to the presence of baseline CKD (defined as eGFR < 60 ml/min/1.73 m^2^), albuminuria, eGFR, or BMI [58].

The third trial DECLARE-TIMI 58 [52] enrolled 17,160 T2DM patients and assessed the effects of dapagliflozin vs. placebo on CV outcomes. The majority of enrolled patients were without an established ACVD (59%). Differently from the previous trials, DECLARE-TIMI 58 had a different design with two co-primary endpoints: MACE (CV death, MI, ischemic stroke) and CV death plus HF hospitalization. Indeed, noticing the unexpected beneficial effect on HF hospitalization both in EMPA-REG outcome and in CANVAS, it was decided to promote HF hospitalization as the primary endpoints. Despite a neutral effect on the MACE outcome, dapagliflozin was significantly superior to placebo in reducing CV death or HF hospitalization with a 17% reduction of the RR (4.7% vs. 5.8%; HR 0.83; 95%, CI 0.73–0.95, *p* = 0.005) [52]. When considered alone, the benefit was significant only for HF hospitalization (RRR 27%; HR 0.73; 95% CI 0.61–0.88) in patient with or without a previous history of HF [52]. In the DECLARE-TIMI 58, the mean eGFR was 85.4 ± 15.8 ml/min/1.73 m^2^ in the dapagliflozin group and 85.1 ± 16.0 ml/min/1.73 m^2^ in the placebo group. An analysis of this trial focused on secondary renal outcome has been reported [60], and it was showed that dapagliflozin reduced the risk of the composite cardiorenal outcome represented by sustained decrease in eGFR by at least 40% and CV or renal death and ESRD by 24% (HR 0.76; 95% CI 0.67–0.87, *p* < 0.001). The subgroup analyses revealed that the effect of dapagliflozin on CV outcomes was similar according to urinary albumin-to-creatinine ratio (UAC) < or > of 30 mg/mmol and to eGFR < or > of 60 ml/min/1.73 m^2^ [52, 60].

Taking into consideration the results from EMPA-REG, CANVAS, and DECLARE-TIMI 58, Zelniker et al. have recently published a meta-analysis [53] on 34,322 patients showing that SGLT2i reduced the risk of renal disease progression by 45% (HR 0.55; 95% CI 0.48–0.64, *p* < 0·0001), with a similar benefit in those with and without established ACVD. Nevertheless, an interaction between baseline renal function and the clinical effects of SGLT2i was noted. The authors divided patients in three groups according to the values of eGFR of less than 60 ml/min/1.73 m^2^, between 60 and 90 ml/min/1.73m^2^, and equal or above 90 ml/min/1.73 m^2^. Reduction of the standardized composite endpoint of renal outcomes including worsening eGFR, ESRD, or renal death was observed across all baseline eGFR values but was more relevant in the group with eGFR > 90 ml/min/1.73 m^2^ [59]. By contrast, the more pronounced risk reduction for HF hospitalization was reported in patients with eGFR values of less than 60 ml/min/1.73 m^2^ [53]. The potential reasons for these results are a topic of active investigation: possibly, the nephroprotective effects induced by SGLT2i and described in our review, in particular the natriuretic effect, could explain the benefits enlightened in patients with worse baseline eGFR, a category at higher risk for HF hospitalization, whereas the greatest reduction of renal composite outcome in those with baseline preserved renal function could be explained by a more pronounced long-term protection of a preserved renovascular structure compared with the protection of SGLT2i obtained in a stage of mild or moderate renal dysfunction, when the damage is only partially reversible. Another recent meta-analysis [61] focused on the effects of both SGLT2i and glucagon-like peptide 1 receptor agonists (GLP1-RA) on CV and renal outcomes. The authors demonstrated that both GLP1-RA (HR 0.82; 95% CI 0.75–0.89; *p* < 0.001) and SGLT2i (HR 0.62; 95% CI 0.58–0.67, *p* < 0.001) reduced the risk of progression of kidney disease including macroalbuminuria, but only SGLT2i reduced the risk of worsening eGFR, end-stage kidney disease, or renal death (HR 0.55; 95% CI 0.48–0.64, *p* < 0.001) [61].

DAPA-HF [5] was the first RCT on SGLT2i dedicated to HF patients irrespective of diabetic status. It enrolled 4744 HFrEF patients to receive dapagliflozin or placebo on top of standard of care therapy for HFrEF. The primary outcome was a composite of CV death plus HF hospitalization or an urgent visit for worsening HF. After a median follow-up of 18.2 months, dapagliflozin significantly reduced the primary EP of 26% (HR 0.74; 95% CI 0.65–0.85, *p* = 0.00001) [5]. The different components of the primary EP were all significantly reduced: CV mortality by 18% (HR 0.82; 95% CI 0.69–0.98, *p* = 0.029) and first hospitalization for HF or urgent visit for HF by 30% (HR 0.70; 95% CI 0.59–0.83, *p* = 0.00003) [5]. Moreover, also all the secondary endpoints were significantly reduced: CV mortality and first hospitalization for HF (HR 0.75; 95% CI 0.65–0.85, *p* = 0.00002), CV mortality and all the hospitalizations for HF (HR 0.75; 95% CI 0.65–0.88, *p* = 0.0002), and all-cause mortality (HR 0.83; 95% CI 0.71–0.97, *p* = 0.022) [5].

The most remarkable result was the reduction of the primary endpoints also in non-diabetic patients, as documented by the prespecified subgroup analyses which did not show any difference regarding the reduction or primary endpoints between diabetic patients (HR 0.75; 95% CI 0.63–0.90) and non-diabetic patients (HR 0.73; 95% CI 0.60–0.88) [5]. In particular, non-diabetic patients had a RR reduction of 27% of the primary endpoint.

Regarding the renal outcome, DAPA-HF enrolled a population with a mean eGFR of 66.0 and 65.5 ml/min/1.73 m^2^ in treated and placebo groups, respectively. However, differently from the previous RCTs, 40.6% and 40.7% in dapagliflozin and placebo groups had an eGFR ≥ 30 and < 60 ml/min/1.73 m^2^, whereas the previous 3 RCTs excluded patients with an eGFR < 60 ml/min/1.73 m^2^. In this population with a more severe CKD, dapagliflozin vs placebo showed no differences regarding the incidence of the secondary endpoint “worsening renal function,” a composite of sustained ≥ 50% reduction in eGFR, ESRD, or death from renal causes (HR 0.71; 95% CI 0.44–1.16, *p* = 0.17) but showed a trend of benefit for dapagliflozin group (worsening renal function occurred in 1.2% of the dapagliflozin group vs 1.6% of controls) [58]. Noteworthy, the prespecified subgroup analysis did not detected any difference regarding the reduction of primary EP in patients with an eGFR ≥ 60 ml/min/1.73 m^2^ with respect to the patient with an eGFR < 60 ml ml/min/1.73 m^2^ [5].

In summary, the previously mentioned 3 RCTs were focused on CVD prevention in T2DM, and they were not originally designed to explore primarily the role of SGLT2i on renal outcome. In fact, they included a limited number of patients with mild CKD, with the consequence of observing a small number of renal events such as dialysis, renal transplantation, or renal death: however, the unexpected magnitude of beneficial effect on secondary kidney outcome gained attention, increasing the expectation for the results of the trials with renal endpoints as primary target.

## The CREDENCE trial

The first of the SGLT2i RCT with a primary cardiorenal composite endpoint was the CREDENCE trial [6]. This trial randomized 4401 patients with TD2M and albuminuric CKD, defined as values of eGFR ≥ 30 and < 90 ml/min/1.73 m^2^ and UAC between 300 and 5000 mg/g, to receive either 100 mg daily of canagliflozin or placebo; all patients were treated with RAAS blockade and 50% had a history of CV disease. The primary outcome was a composite endpoint of ESRD (defined as dialysis, transplantation, or a sustained eGFR less than 15 ml/min/1.73 m^2^), a doubling of the serum creatinine level, or death from renal or cardiovascular cause.

The trial was stopped early, with a median follow-up of 2.6 years, after a planned ad interim analysis. At that time, the event rate of the primary endpoint was significantly reduced in the canagliflozin group compared with placebo, with a RR reduction of 30% (HR 0.70; 95% CI 0.59–0.82, *p* = 0.00001) [6]. In addition, patients randomized to canagliflozin presented a significant RRR of 34% (HR 0.66; 95% CI 0.53–0.8, *p* < 0.001) for the renal composite outcome of ESRD, doubling of the serum creatinine level, or renal death [6]; the nephroprotective role of canagliflozin was consistent also for the single components of composite endpoint ESRD (HR 0.68; 95% CI 0.54–0.86, *p* = 0.002) and for the exploratory outcome of dialysis, kidney transplantation, or renal death (HR 0.72; 95% CI 0.54 to 0.97) [6].

Once more, after the first weeks of treatment, there was an initial greater reduction of eGFR in the canagliflozin than in the placebo group (− 3.72 ± 0.25 vs. − 0.55 ± 0.25 ml/min/1.73 m^2^; 95% CI, − 3.87 to − 2.47) [6]; afterwards, the reduction of eGFR was slower in the canagliflozin group than in the placebo group (− 1.85 ± 0.13 vs. − 4.59 ± 0.14 ml/min/1.73 m^2^ per year; 95% CI, 2.37 to 3.11) [6]. Several secondary CV outcomes were tested in a hierarchical fashion: canagliflozin reduced the relative risk of the composites of CV death or hospitalization for HF (HR 0.69; 95% CI 0.57.0.83, *p* < 0.001), CV death, myocardial infarction, or stroke (HR 0.80; 95% CI 0.67–0.95, *p* = 0.01), and hospitalization for HF(HR 0.61; 95% CI 0.47–0.80, *p* < 0.001) [6]. These findings strongly support the concept that canagliflozin may have clear and relevant effects for both renal and CV protection in patients with T2DM and CKD; furthermore, the authors suggested that the benefits observed in the CREDENCE trial were also consistent regardless of baseline diuretic use, different levels of hypertension or glucose control, and weight loss [62].

## Conclusions

Despite that SGLT2i were originally thought and developed as glucose-lowering drugs, unexpectedly, they documented a significative reduction of death and CV events even in the absence of diabetes and demonstrated a cardiorenal protective effects. Whereas different trials mainly focused on prevention of CV outcomes in TD2M patients, there was a clear benefit on HF that was initially explored only as secondary endpoint. Later on, the DAPA-HF trial proved a clear benefit on CV mortality and worsening HF both in diabetic and in non-diabetic patients but also in patients with a moderate to severe CKD. Likewise, there was an unforeseen benefit in renal outcomes—initially evaluated only in patients with a mild CKD—that taken together with HF hospitalization dragged most of the benefit in the 3 RCTs dedicated to CVD prevention [50–52].

The CREDENCE trial demonstrated the role of canagliflozin in reducing the risk of kidney failure, analyzed as primary outcome, and CV events in patients with T2DM and CKD, and thanks to the CREDENCE trial, the European Medicines Agency’s (EMA) Committee for Medicinal Products for Human Use (CHMP) has adopted a positive opinion to extend the indication of canagliflozin (at the lower dose of 100 mg day) to include important renal outcomes.

In line with the CREDENCE trial, in patients with T2DM post hoc analysis [63] of two registration trials with ertugliflozin, a new drug in the class with high selectivity for SGLT2, confirmed that over 104 weeks of treatment, eGFR values were higher and UAC reduced compared with non-SGLT2i treatment, even though changes in HbA1c did not differ between the groups; with respect to this drug, data from the CV safety VERTIS trial, recently presented at the American Diabetes Association, including secondary composite renal endpoints are eagerly awaited.

The effects in the kidney derived from SGLT2 inhibition are likely to be multifactorial, with different direct and indirect mechanisms. Additional studies will clarify the potential clinical role of SGLT2i on kidney function in patients without T2DM: at this stage, we have data generated following a very short period of time (6 weeks) of treatment with dapagliflozin, the DIAMOND trial [64], in which patients with non-diabetes-related CKD did not show a beneficial effect on proteinuria when compared with the placebo group. The effects of a longer period of treatments are warranted.

In the meantime, taking into consideration the bidirectional nature of the heart-kidney interplay, the susceptibility of T2DM patients to develop HF and CKD, and the results of the CVD safety trails, of the CREDENCE trial and of DAPA-HF, we believe that this class of drugs has already become a milestone therapy for cardiorenal protection in patients with T2DM and in patients with HF.
